# Machine learning methods for gene regulatory network inference

**DOI:** 10.1093/bib/bbaf470

**Published:** 2025-09-18

**Authors:** Akshata Hegde, Tom Nguyen, Jianlin Cheng

**Affiliations:** Department of Electrical Engineering and Computer Science, University of Missouri, 416 S 6th St, Columbia, MO 65201, United States; Roy Blunt Nextgen Precision Health, University of Missouri, 1030 Hitt St, Columbia, MO 65205, United States; Department of Electrical Engineering and Computer Science, University of Missouri, 416 S 6th St, Columbia, MO 65201, United States; Roy Blunt Nextgen Precision Health, University of Missouri, 1030 Hitt St, Columbia, MO 65205, United States; Department of Electrical Engineering and Computer Science, University of Missouri, 416 S 6th St, Columbia, MO 65201, United States; Roy Blunt Nextgen Precision Health, University of Missouri, 1030 Hitt St, Columbia, MO 65205, United States

**Keywords:** machine learning, gene regulatory network, GRN, inference, deep learning, omics

## Abstract

Gene Regulatory Networks (GRNs) are intricate biological systems that control gene expression and regulation in response to environmental and developmental cues. Advances in computational biology, coupled with high-throughput sequencing technologies, have significantly improved the accuracy of GRN inference and modeling. Modern approaches increasingly leverage artificial intelligence (AI), particularly machine learning techniques—including supervised, unsupervised, semi-supervised, and contrastive learning—to analyze large-scale omics data and uncover regulatory gene interactions. To support both the application of GRN inference in studying gene regulation and the development of novel machine learning methods, we present a comprehensive review of machine learning-based GRN inference methodologies, along with the datasets and evaluation metrics commonly used. Special emphasis is placed on the emerging role of cutting-edge deep learning techniques in enhancing inference performance. The major challenges and potential future directions for improving GRN inference are also discussed.

## Introduction

Gene expression is the process by which genetic information synthesizes functional products, such as RNA and proteins, and is critical in all living organisms [1]. Proper regulation of gene expression is essential to ensure that genes are activated only when necessary and that their activity is properly controlled [2]. The regulation of gene expression is achieved through understanding the intricate interactions between genes and other molecules. In this effort, Gene Regulatory Networks (GRNs) have emerged as a strong tool [3].

GRNs are complex systems that determine the development, differentiation, and function of cells and organisms, as well as their response to environmental stimuli [4, 5]. GRNs consist of genes, transcription factors (TFs), microRNAs, and other regulatory molecules that interact with each other to control gene expression [6]. The regulatory interactions between these molecules can form complex networks that exhibit emergent properties, such as robustness and adaptability [7]. In its simplest form, a GRN is a network of genes and their regulatory interactions, which govern the expression of these genes in response to various cellular cues. It is worth noting that in this definition, a TF is considered a special kind of gene that may regulate the expression of other nonTF or TF genes. Each gene in the network acts as a node, and the regulatory interactions between genes are represented by directed edges connecting these nodes [8].

**Figure 1:**
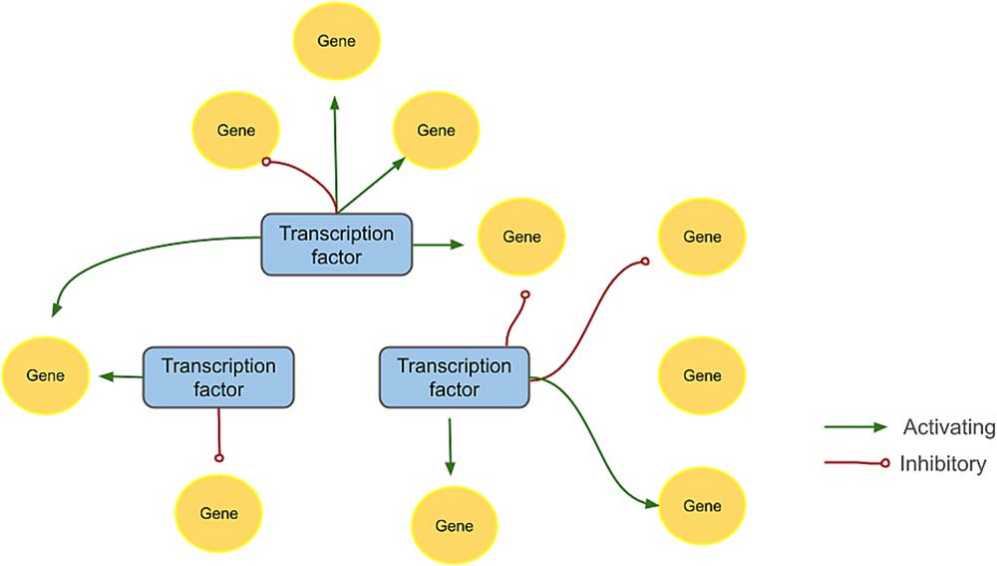
Simple gene regulatory network. Circular nodes represent genes, and rectangular nodes represent transcription factors. Regulatory interactions are indicated by edge style: Solid arrows represent activation, while lines ending with a circular blunt symbol represent inhibition.

Fig. 1 illustrates a simple GRN [9]. Interactions (edges) can activate or repress, forming networks that regulate gene expression across cellular states and environmental conditions. GRN topology evolves through duplication, mutation, and selection, giving rise to novel regulatory mechanisms [4]. Computational and experimental analyses reveal how GRNs govern signaling, gene regulation, and protein interactions [4, 6], with implications for systems biology, developmental biology, cancer research, evolutionary studies, and personalized medicine [4].

GRN inference or modeling is the process of identifying these interactions among genes that contribute to the regulation of gene expression. Over time, the study of GRNs has evolved from the early days of molecular biology to the current era of computational biology due to the generation and accumulation of huge amounts of multi-omics (e.g. genomics and transcriptomics) data that can be used to infer underlying gene regulation mechanisms.

The study of GRNs has a rich history that dates to the early days of molecular biology, when researchers first began to uncover the basic principles of gene regulation, such as the role of TFs in controlling gene expression [10]. In the late 1980s and 1990s, techniques such as DNA foot printing [11] and electrophoretic mobility shift assays (EMSAs) [12] were developed to identify TF binding sites in DNA sequences [13, 14]. The advent of microarray technology in the early 2000s allowed for large-scale studies of gene expression patterns [15], which paved the way for more advanced GRN modeling techniques.

High-throughput multi-omics experiments proliferated in the last two decades—RNA-seq for high-resolution expression [16, 17], single-cell sequencing for cellular heterogeneity [18], ChIP-seq for TF binding [19], and ATAC-seq for chromatin accessibility [20]—have transformed GRN inference but also increased data complexity, driving the need for robust AI-driven models and standardized benchmarks like the DREAM challenges on *Escherichia coli* and *Saccharomyces cerevisiae* [21–23]. Classic machine learning methods (e.g. Bayesian networks, Random Forests, support vector machines (SVMs), gradient boosting, logistic regression, and neural nets) laid the groundwork, achieving moderate accuracy [24, 25], but deep learning now leads the field by modeling complex, nonlinear regulatory relationships, and surpassing clustering-based methods [26]. Although prior reviews have covered early computational strategies [27], transcriptomics-only approaches [28], and chromosome-structure methods [29], there remains no single synthesis that integrates the latest deep-learning advances across these diverse data modalities.

In this review, we aim to fill that gap by systematically categorizing state-of-the-art machine learning approaches for GRN inference, with a particular emphasis on the latest deep learning models. Unlike previous reviews, we not only classify methods based on algorithmic approaches, but also consider the types of data sources (e.g. transcriptomics, epigenomics, and chromatin structure) and the specific forms of GRN inference they enable. This multidimensional framework is intended to provide researchers with a clearer understanding of current trends, emerging challenges, and future opportunities in the field.

## Machine learning methods for gene regulatory network inference

We categorize GRN inference methods broadly based on the type of machine learning methods, i.e. supervised learning, unsupervised learning, semi-supervised learning, and contrastive learning methods.

Table 1 provides an overview of a list of various GRN inference algorithms categorized by their learning paradigms (supervised, unsupervised, semi-supervised, and contrastive learning), utilization of deep learning techniques, compatibility with bulk/single cell RNA seq data, year of publication, and the core computational technologies employed. The list includes 14 recent deep learning methods developed in the last five years as well as nine other typical non-deep learning machine learning methods for GRN inference. We focus on reviewing the recent representative deep learning methods, while considering some nondeep learning methods to provide a broad perspective of the field. Moreover, many additional methods that extend some popular methods in Table 1 but are not listed there will also be discussed.

**Table 1:** The categorization of 23 recent or representative machine learning methods for GRN inference.

**Algorithm name**	**Learning type**	**Deep learning**	**Input type**	**Year**	**Key technology**	**Link**
GENIE3	Supervised	No	bulk	2010	Random forest	https://github.com/vahuynh/GENIE3
SIRENE	Supervised	No	bulk	2009	SVM	http://cbio.ensmp.fr/sirene
GRADIS	Supervised	No	Single-cell	2023	Support vector machine	https://github.com/MonaRazaghi/GRADIS
DeepIMAGER	Supervised	Yes	Single-cell	2024	CNN	https://github.com/shaoqiangzhang/DeepIMAGER
DeepSEM	Supervised	Yes	Single-cell	2023	Deep structural equation	https://github.com/HantaoShu/DeepSEM
STGRNs	Supervised	Yes	Single-cell	2023	Transformer	https://github.com/zhanglab-wbgcas/STGRNS
RSNET	Supervised	Yes	Single-cell	2022	Graph convolutional net	https://github.com/zhanglab-wbgcas/rsnet
dynGENIE3	Supervised	No	Single-cell	2018	Random Forest modeling	http://www.montefiore.ulg.ac.be/ huynh-thu/dynGENIE3.htm
GRNFormer	Supervised	Yes	Single-cell	2025	Graph Transformer	https://github.com/BioinfoMachineLearning/GRNformer.git
AnomalGRN	Supervised	Yes	Single-cell	2025	Graph anomaly detection	https://github.com/ZZCrazy00/AnomalGRN
LASSO	Unsupervised	No	Bulk	2016	Regression	https://github.com/omranian/inference-of-GRN-using-Fused-LASSO
ARACNE	Unsupervised	No	bulk	2006	Information theory	https://califano.c2b2.columbia.edu/aracne
MRNET	Unsupervised	No	bulk	2007	Min. redundancy/info theory	https://bioconductor.org/packages/release/bioc/html/minet.html
BiGRN	Unsupervised	Yes	bulk	2022	Bidirectional RNN	https://gitee.com/DHUDBLab/bi-rgrn
CLR	Unsupervised	No	bulk	2007	Mutual information	https://bioconductor.org/packages/release/bioc/html/minet.html
GENECI	Unsupervised	No	bulk	2023	Evolutionary ML	https://github.com/AdrianSeguraOrtiz/GENECI
CVGAE	Unsupervised	Yes	Single-cell	2024	Graph neural Network	None
GRN-VAE	Unsupervised	Yes	Single-cell	2020	Variational autoencoder	https://bcb.cs.tufts.edu/GRN-VAE
BiRGRN	Unsupervised	Yes	Single-cell	2022	Bidirectional RNN	https://gitee.com/DHUDBLab/bi-rgrn
DeepMAPS	Unsupervised	Yes	scATAC/Multi-omic	2023	Heterogeneous graph transformer	https://github.com/OSU-BMBL/deepmaps
GRGNN	Semi-Supervised	Yes	Single-cell	2020	Graph neural network	https://github.com/juexinwang/GRGN
GCLink	Contrastive	Yes	Single-cell	2025	Graph contrastive link prediction	https://github.com/Yoyiming/GCLink
DeepMCL	Contrastive	Yes	Single-cell	2023	CNN	https://github.com/lzesyr/DeepMCL

The diversity of approaches listed highlights the evolution of GRN modeling from classical machine learning methods (e.g. Random Forests, SVMs) to more recent deep learning frameworks including convolutional neural networks (CNNs), variational autoencoders (VAEs), graph neural networks (GNNs), and graph transformers.

### Supervised learning methods for gene regulatory network inference

Supervised learning is a fundamental approach in machine learning where algorithms are trained on labeled datasets—i.e. datasets in which each input is paired with a known output. By analyzing these labeled examples, the algorithm learns to recognize patterns and relationships between inputs and their corresponding outputs. Once trained, the model can generalize this knowledge to make predictions on new, unseen data [30]. In the context of GRN inference, supervised learning enables the prediction of direct downstream targets of TFs by leveraging labeled datasets containing experimentally validated regulatory interactions. This approach allows models to learn from known gene-regulatory relationships and apply that knowledge to uncover novel interactions with improved accuracy [31].

**Figure 2:**
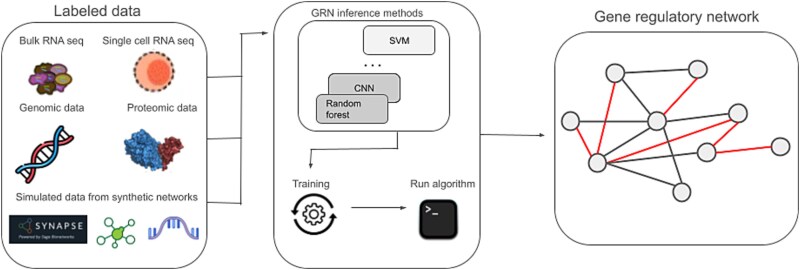
Gene regulatory network training and inference framework. Various labeled data sources (RNA-seq, genomic, proteomic, or synthetic data) can be integrated with machine learning methods such as SVM, CNN, random forest or other deep learning models to train and infer gene regulatory networks. Circular nodes represent genes; edges indicate regulatory interactions, either activation or inhibition.

Fig. 2 depicts the supervised GRN inference training–test pipeline, using algorithms such as random forests, SVMs, regression, and deep learning. When sufficient labeled interactions exist, supervised methods outperform unsupervised and semi-supervised approaches [32, 33], but accuracy declines with scarce labels. The following section reviews key supervised learning techniques and highlights recent advancements in deep learning approaches for GRN reconstruction.

Random forest ensembles are a mainstay of supervised GRN inference. GENIE3 formulates GRN prediction as a set of regression tasks, where each gene’s expression is modeled using Random Forest or Extra Trees algorithms, based on the expression of all other genes. The resulting variable importance scores are aggregated into a genome-wide ranking of regulatory interactions. This method performed exceptionally well on both synthetic and real datasets, winning the DREAM4 *In Silico* Multifactorial challenge, and remains a state-of-the-art classic approach [34].

dynGENIE extends GENIE3 with a semi-parametric framework based on ordinary differential equations (ODEs), where a nonparametric forest learns transcription functions that integrate time-series and steady-state data [35]. Variants like GENIMS use guided forests and *q*-norm weight normalization to enhance precision [21], while GENIE3-time incorporates time-lagged interactions, and BTNET replaces bagging with boosted trees that iteratively correct weak estimators for improved accuracy [22, 23]. Additional forest-based methods include iRafNET, GENREF, and GRRFNet [36–38]. While TIGRESS also applies tree ensembles, GENIE3 and its derivatives consistently outperform it in benchmark evaluations [39].

Another widely used supervised learning algorithm in GRN inference is the SVM, which is well-suited for binary classification tasks [40]. In this context, SVM leverages kernel functions to compute pairwise similarities between genes and identifies a hyperplane that optimally separates regulatory from nonregulatory interactions in the feature space [41]. A pioneering application of SVM to GRN inference is SIRENE, which integrates gene expression data with known TF–target gene relationships [42]. SIRENE reformulates the global network inference task as a series of local binary classification problems, training an individual SVM for each TF to distinguish between its regulated and nonregulated targets. The underlying assumption is that co-regulated genes exhibit similar expression profiles. Empirical evaluations show that SIRENE outperforms several unsupervised methods in specific settings. CompareSVM is another software tool that predicts GRNs from expression data using a SVM. It optimizes parameters, compares kernel accuracy by generating AUC (area under the curve) for each kernel, and selects the best-performing kernel method for prediction [43]. One of the recent methods, the GRADIS approach, uses SVMs to reconstruct gene-regulatory networks by providing feature vectors based on graph distance profiles from a network representation of the gene expression data. It was shown to outperform existing supervised approaches on the synthetic data and two benchmark datasets of *E. coli* and *S. cerevisiae* provided by the DREAM4 and DREAM5 network inference challenges [44]. Many other methods use supervised learning that uses the SVM approach, like Beacon GRN [45], and supervised ensemble approaches like EnGRaiN [46] for GRN inference.

RSNET is another supervised learning method that uses an information constraint-based approach to infer GRNs. It constrains candidate genes with highly dependent parameters measured from the data by mutual information (MI) as network enhancement items and highly putative candidate regulators as supervisors to improve optimization efficiency [47]. A lot of GRN inference tools also are developed on regression-based methods like Least Square Cut-Off (LSCO) [48], LASSO [49], and Ridge-regression with Cut-Off (RidgeCO) [50]. Least Square Cut-Off with Normalization (LSCON) [51] is one of the latest methods i.e. built on LSCO [52].

Recent advances in supervised deep learning have created significant waves in the field of technology and are now being implemented in various domains, including GRN inference. One of the most popular supervised neural network architectures is the CNN [53]. DeepIMAGER [54] uses the ResNet50 CNN to infer GRNs and employs a supervised approach that converts the co-expression patterns of gene pairs into an image-like representation while incorporating improved TF binding information for training. The dataset used in the study comprises single-cell RNA-seq (scRNA-seq) and ChIP-seq data, which capture TF–gene pair information across different cell types. It was shown that DeepIMAGER outperforms existing methods such as GENIE3 [34], PIDC [55], SCODE [56], PPCOR [57], and SINCERITIES [58] in some experiments.

Another supervised deep learning method for GRN inference is SPREd. SPREd [59] is a simulation-supervised neural network whose data include expression relationships among targets and between TFs within TF pairs. The model is trained using synthetic gene expression data produced by a simulation framework inspired by biophysical principles. This framework integrates both linear and nonlinear TF–gene interactions and simulates various GRN architectures. It was shown that SPREd performs better than other state-of-the-art models, such as GENIE3 [34], ENNET [60], PORTIA [61], and others in some experiments, particularly on datasets with strong co-expression among TFs.

Recently, a cutting-edge deep learning technology, transformer, has emerged as a powerful deep learning architecture, particularly for modeling complex interactions among genes and TFs. Originally designed for natural language processing tasks, the self-attention mechanism in transformers enables them to capture long-range dependencies, leading to greater efficiency for GRN inference—especially when gene interactions are complex and span multiple regulatory layers. When applied to GRNs, transformers can model how one gene regulates another across various time points or conditions, even when these relationships are nonlinear or occur over long distances. A key benefit of using transformers is their ability to work well with high-dimensional datasets, such as transcriptomic data, and to accurately construct gene interaction networks. For instance, research has applied transformer-based models to gene expression data, demonstrating that these models outperform traditional methods by capturing sequential and structural relationships within GRNs [62–64].

One tool that employs transformers is STGRNs. STGRNs consist of four components: the GEM module, the positional encoding layer, the transformer encoder, and the classification layer [65]. The GEM module converts gene pairs into a format suitable for input into the transformer encoder. The positional encoding layer extracts positional or temporal information. The transformer encoder computes the relationships among various subvectors, and the classification layer then makes the final categorization of the outcome. Results obtained with scRNA-seq data indicate that STGRNs outperform comparable tools in some experiments and are more interpretable.

One of our latest works, GRNFormer [66] is an end-to-end deep learning framework that infers GRNs from single-cell RNA-seq data using a variational graph transformer autoencoder. It identifies relationships such as co-expression patterns and TF influences across different biological contexts and species.

**Figure 3:**
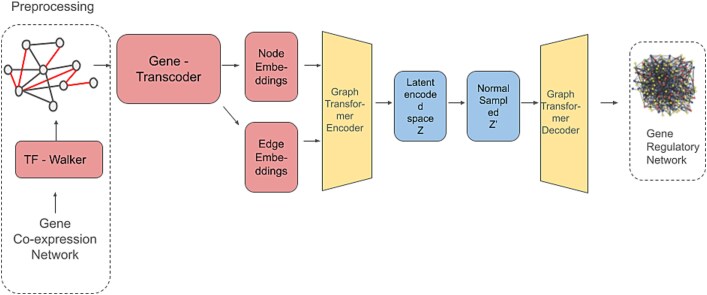
Architecture diagram of GRNFormer [66] for gene regulatory network inference. A gene co-expression network is first preprocessed with TF-Walker. Gene-transcoder generates node and edge embeddings, which are encoded by a graph transformer into a latent space. After sampling, the embeddings are decoded by a graph transformer decoder to reconstruct the gene regulatory network.

Fig. 3 show the pipeline process of GRNFormer which begins with the construction of a gene co-expression network (GCEN). GRNFormer starts by normalizing gene expression data using ArcSinh, then constructs a gene co-expression network (GCEN) based on Pearson correlation, keeping only significant gene–gene associations. To handle high dimensionality, it uses a **TF-Walker** algorithm, which samples subgraphs centered on TFs by selecting nearby genes until a fixed size (100 nodes) is reached. Z-score normalization is applied within each subgraph to standardize expression ensuring that local expression contexts are accurately captured while reducing computational burden.

These subgraphs are processed by the GENE-Transcoder, which uses a 1D convolution to capture local patterns and transformer encoder layers with multi-head attention for both local and global interactions; mean pooling then produces compact, context-aware gene embeddings. The embeddings, together with GCEN edge features, pass through a variational graph transformer autoencoder that computes pairwise attention scores and models uncertainty using a Gaussian latent distribution. The decoder refines node embeddings, generates edge attention scores, and produces a probabilistic adjacency matrix via an inner product and sigmoid activation; aggregated sub networks form the full GRN. Training employs ground truth regulatory data (e.g. ChIP-seq, STRING) with a composite loss function (binary cross-entropy plus Kullback–Leibler divergence) and dynamic negative sampling to address class imbalance.

AnomalGRN [67] reframes GRN inference as graph anomaly detection. It converts every gene pair into a node whose feature vector combines their expression and regulatory signals, then uses cosine similarity to group homogeneous nodes and flag heterogeneous “anomalies” as candidate regulatory links. Graph sparsification prunes noisy, redundant edges typical of single cell RNA-seq, clarifying structure before anomaly scoring. Tested on multiple single cell benchmarks, AnomalGRN outperformed other methods: it reliably recovered known interactions and revealed novel hub genes and TF–target pairs, showing that treating rare regulatory events as anomalies can overcome severe class imbalance and dropout noise.

### Unsupervised learning for gene regulatory network inference

Unsupervised approaches are critical when validated TF–gene pairs are limited, as they uncover latent regulatory patterns in large gene expression compendia. Evolutionary machine learning (EML) couples genetic-algorithm operators—selection, mutation, crossover—with standard learners to optimize network topology via a fitness function that rewards sparsity, coherence, and agreement with expression statistics [68]. GENECI builds on this by clustering edge lists from multiple inference methods into a consensus network and then evolving it to maximize topological quality and confidence while penalizing contradictions. On the DREAM challenges and the IRMA benchmark, GENECI produced stable, high-precision GRNs and even pinpoints melanoma-relevant regulators, demonstrating the clinical promise of EML-driven consensus strategies [69].

A second unsupervised pillar is information theory [70]. Mutual Information (MI) measures how much knowledge of one gene’s expression reduces uncertainty about another, capturing both linear and nonlinear dependencies without requiring prior labels and scaling to thousands of genes. Practical MI estimation for continuous, noisy data relies on adaptive binning, kernel density or k nearest neighbor methods; each must balance bias and variance, and results are sensitive to sample size, correlation strength and distributional shape. **ARACNE** mitigates MI’s tendency to retain indirect associations by invoking the data processing inequality: after computing pairwise MI scores, it discards edges explainable through a higher MI intermediary, producing a sparser, more biologically credible network that performs well in genome wide studies where indirect correlations abound [71]. The algorithm has been applied to tumor expression cohorts to map oncogenic circuitry and to developmental time courses to isolate stage specific regulators, demonstrating how information theoretic pruning can translate raw co-expression into actionable biological insight.

Similarly, MRNET applies the minimum redundancy–maximum relevance (mRMR) criterion to rank regulators: it maximizes MI with each target while penalizing redundant predictors, clarifying true signals in noisy, high-dimensional data and performing competitively on 30 synthetic microarray benchmarks [72]. CLR builds on ARACNE by comparing every MI score to its empirical background; interactions that rise above this context are retained, yielding networks more resilient to variability and measurement noise.

While MRNET and CLR rely on pairwise information, newer unsupervised deep learning models exploit temporal structure and nonlinear patterns that classical metrics cannot capture. Bidirectional RNNs read expression time series in both forward and reverse directions, integrating past and future dependencies; BiRGRN embeds this architecture in an unsupervised framework and outperforms earlier tools on four simulated and three real scRNA-seq datasets [73]. VAEs further generalize unsupervised inference by compressing high dimensional profiles into latent spaces that preserve key regulatory structure [74]; GRNVAE leverages these representations to uncover subtle, nonlinear TF–target links that linear models overlook, providing richer views of gene regulation in large, heterogeneous datasets [75].

DeepMAPS [76] extends graph attention mechanism to GRN inference by coupling a heterogeneous graph transformer (HGT) with single cell multi-omics data. After filtering low quality cells/genes, normalizing each modality and integrating them into a unified cell–gene matrix, DeepMAPS constructs a heterogeneous graph whose nodes are cells and genes and whose edges denote gene occurrence within cells. GNN autoencoders compress this matrix, then an HGT jointly refines cell and gene embeddings while producing attention scores that quantify each gene’s importance to each cell. These scores drive the simultaneous discovery of cell clusters and functional gene modules. To infer GRNs, DeepMAPS seeds regulons from Reactome, Dorothea and TRUST v2, then uses the learned attention patterns to assemble cell type specific networks that capture stimulus responsive, active biology. Benchmarking against IRIS3 on scRNA-seq, scATACseq and combined inputs shows higher accuracy and interpretability, confirming DeepMAPS’ utility for uncovering key regulators and pathways. Despite these strengths, DeepMAPS has practical constraints. Graphs with millions of cells or billions of edges tax its memory and runtime, GPU execution is recommended but can yield slightly different results across hardware owing to floating point precision, and performance drops when data are noisy or suffer batch effects. Nonetheless, by integrating auto encoder derived embeddings with HGT attention, DeepMAPS offers an end to end, transformer based framework for high resolution, cell type specific network reconstruction.

Additionally, DeepSEM (deep structural equation modeling) [77] represents a hybrid approach that combines the strengths of structural equation modeling (SEM) with deep learning methods. SEM is a statistical technique typically used to model relationships between observed and latent variables, which makes it suitable for GRN inference when direct relationships among genes are not readily observable. DeepSEM extends this framework by incorporating deep learning to capture both linear and nonlinear dependencies, resulting in a flexible and robust method for inferring GRNs in complex biological systems.

Recently, another deep learning designed for graph-structured datasets called GNNs was also applied to GRN inference. Unlike traditional neural networks, which process Euclidean data such as images or text, GNNs excel at analyzing non-Euclidean data, where relationships among entities are represented as nodes connected by edges in a graph. GNNs enhance both the representation of individual node features and the overall structure of graphs, making them well-suited for applications such as social network analysis, molecular structure prediction, and GRN inference [78]. For example, CVGAE [79] applies a GNN that combines gene expression data with network topology to embed the data into a low-dimensional vector space. This vector is then used to compute distances between genes and predict interactions. CVGAE employs multistacked GraphSAGE layers as the encoder and an enhanced decoder to address network sparsity. Evaluations on various single-cell datasets—including four ground-truth networks—indicate that CVGAE performs exceptionally well compared with other tools.

### Semi-supervised learning for gene regulatory network inference

Semi-supervised learning sits between supervised and unsupervised approaches by exploiting a small set of experimentally validated edges together with abundant unlabeled expression profiles—an attractive compromise when regulatory labels are costly to obtain. In this setting, labeled interactions steer the model toward biologically plausible solutions, while unlabeled data broaden coverage and reduce overfitting.

TSNI (time-series network inference) exemplifies this strategy for temporal data. It fits a dynamical-systems model to time-series expression, using known edges as anchors and the remaining trajectories as unlabeled input. By iteratively adjusting model parameters, TSNI recovers direct, causal links that explain how gene activities propagate through time, making it valuable for studies of differentiation, circadian control, and environmental responses [80].

Optimization-based schemes add a different twist. Genetic algorithms (GAs) encode candidate GRNs as chromosomes and evolve them under selection; fixed labeled edges serve as hard constraints, while unlabeled data guide fitness evaluation. A GA combined with an SVM for fixed size subset selection demonstrated strong performance on both simulated and real datasets, reliably pinpointing optimal regulator sets for each TF [81, 82].

Pushing deeper into representation learning, GRGNN (Graph Recurrent Gene Neural Network) unifies GNNs with recurrent units. It constructs a gene graph, propagates information through GNN layers to capture topology, and models temporal dynamics with RNNs. Verified edges provide partial supervision, whereas the bulk of unlabeled expression data refine hidden states across time steps. This hybrid design allows GRGNN to infer complex, temporally dependent regulation in networks with intricate connectivity patterns [83].

### Contrastive learning for gene regulatory network inference

Contrastive learning for GRNs. Self-supervised contrastive learning embeds high-dimensional expression data so that gene pairs sharing regulatory roles cluster, whereas unrelated pairs repel. Models construct positive pairs—often two augmented views of the same TF–target pair—and negative pairs of unconnected genes, then optimize an InfoNCE loss that maximizes similarity within positives and minimizes it across negatives; temperature scaling sharpens this margin [84, 85, 86].

DeepMCL. This framework applies multi-view contrastive learning to heterogeneous single cell RNA-seq [87]. Each TF–gene candidate is converted to a histogram of binned co-expression values, and paired histograms pass through a Siamese VGGstyle CNN with nonlocal blocks to learn low-dimensional embeddings. An attention module fuses views from different platforms, time points, and neighboring genes, accentuating informative signals while damping noise and dropout. Concatenated embeddings feed a fully connected classifier that achieves high accuracy on synthetic and real datasets, reducing false positives typical of single cell noise.

GCLink. Extending the idea to graph scale, GCLink frames GRN reconstruction as contrastive link prediction [88]. Observed regulatory edges serve as positives, random non edges as negatives; graph augmentations (node dropout, edge perturbation) create diverse views. A graph encoder learns embeddings that preserve both local and global topology, aligning positive edges, and separating negatives. This approach improves network resolution and robustness to noise by fully exploiting inherent graph structure.

## Types of Inputs and Outputs of gene regulatory network Inference

### Types of outputs of gene regulatory network inference

GRN Inference methods can be classified into groups according to the output that they produced: (i) local GRN inference methods and (ii) global GRN inference methods.

Local GRN inference targets a single gene or a small gene set, modelling direct regulator–target links with focused, high quality data. Methods usually fit statistical or machine learning models that relate a candidate regulator’s expression to that of its putative target. Inferelator applies sparse regression with stability selection to pick the TFs that best predict each target’s expression, yielding compact, and interpretable subnetworks [89]. MRNET instead measures pairwise MI and applies a maximum relevance/minimum redundancy filter to retain the strongest, most independent dependencies, efficiently detecting direct interactions though it captures little of the surrounding network context [90].

Global GRN inference seeks a genome-wide map, integrating both direct and indirect relationships across thousands of genes. These approaches must cope with high-dimensionality and experimental noise typical of bulk RNA-seq, single cell RNA-seq, or GTEx scale datasets. *GENIE3* treats every gene as a regression problem solved by random forests, ranking regulators by their importance scores and assembling them into a full network [34]. *ARACNE* also begins with mutual information but applies the data processing inequality to prune indirect edges, sharpening the final network representation [71]. Such global maps underpin studies of development, disease, and environmental response by providing a systems level view of regulation.

Once a network is inferred, visualization and enrichment analysis are critical for biological interpretation. *Cytoscape* offers an opensource, plug in rich platform that imports GRN edge lists, overlays expression or functional annotations, and supports topology metrics, clustering, and pathway enrichment through community “apps.” Customizable layouts and node/edge styling make it straightforward to highlight key regulators, modules, or condition specific changes, turning complex GRNs into actionable insights [91].

### Types of input data for gene regulatory network inference

GRN inference methods usually take some high-throughput omics data as input to infer GRNs. Different GRN inference methods may work with different types of data. The most commonly used data are genomics and transcriptomics data because of their near universal availability, while other omics data can also provide complementary information if available. Below are some major data sources that can be leveraged for GRN inference.

- Genomic data provide full DNA sequences, regulatory elements, and variants needed to locate TF binding regions [92]. Projects such as 1000 Genomes and ICGC add population scale variant catalogs that reveal how genetic changes reshape GRNs.
- Transcriptomic data quantify RNA abundance via RNA-seq, powering GRN tools like ARACNe and GENIE3 [34, 71]. Repositories—GEO, ArrayExpress, and GTEx—supply vast expression compendia for large-scale reconstructions.
- Epigenetic data (e.g. DNA methylation, histone marks) refine GRNs by flagging active regulatory regions; ChIP-seq profiles protein–DNA contacts [93], while ENCODE and Roadmap Epigenomics curate diverse epigenomic maps. Such datasets remain sparser—and less exploited—than genomic or transcriptomic resources.
- Proteomic data capture protein abundance and posttranslational states, exposing posttranscriptional control [94]. Mass spectrometry efforts like CPTAC link these profiles to genomes, yet proteomic inputs are still relatively scarce and underused in GRN modeling.
- Single-cell multi-omics simultaneously measure, e.g. RNA expression and chromatin accessibility, enabling cell specific GRNs [95, 96]; integrative frameworks such as Seurat [97] and MOFA [98] harness this heterogeneity.
- Gene expression + PPI networks combine transcript levels with physical interaction evidence from STRING or BioGRID; integrative methods like PANDA use these datasets to sharpen regulatory predictions [99].

Table 2 lists principal data repositories for GRN inference. Open resources—ENCODE, GEO, TCGA, GTEx, Roadmap—provide vast expression and epigenomic data, whereas licensed databases like TRANSFAC and Oncomine add curated regulatory knowledge. Merging expression profiles with epigenomic or multi-omics layers from these sources yields more accurate, context specific GRNs because each dataset highlights a distinct regulatory facet.

**Table 2:** A list of major data sources for GRN inference.

**Dataset name**	**Omics type**	**Source**	**Description**
ENCODE	Epigenomic	NIH	Catalogs TF binding, chromatin marks, and regulatory elements.
TCGA	Multi-omics	NCI	Cancer genomic data (DNA, RNA, epigenetics, proteomics) for over 30 tumor types.
ICGC	Multi-omics	International consortium	Global effort sequencing 50+ cancer types (genomic, transcriptomic, epigenomic).
GTEx	Transcriptomic/genomic	NIH	Tissue-specific expression data linked with donor genotypes (eQTLs).
Roadmap Epigenomics	Epigenomic	NIH	Reference epigenomes (histone marks, DNA methylation) across diverse human tissues.
BLUEPRINT	Epigenomic	EUFP7 (BLUEPRINT)	Epigenomes of blood cells (histone marks, methylation).
GEO	Transcriptomic	NCBI	Repository for functional genomics data (RNA-seq, microarray).
ArrayExpress	Transcriptomic	EMBL-EBI	Functional genomics archive, overlapping with GEO.
CCLE	Multi-omics	Broad / Novar-tis	Data for 1000 cancer cell lines (genomics, expression, drug response).
LINCS L1000	Transcriptomic	NIH LINCS /Broad	Large perturbation dataset (1 M profiles) capturing expression changes.
Human Cell Atlas	Multi-omics	HCA Consor-tium	Single-cell data (RNA, ATAC) from various human tissues.
Cistrome DB	Epigenomic	X. Liu Lab	Curated ChIP-seq/ATAC-seq for TF binding and chromatin accessibility.
TRANSFAC	Genomic / Regulatory	geneXplain	TF binding motifs and consensus sites (license required).
Oncomine	Transcriptomic	Thermo Fisher	Cancer gene expression platform with curated datasets.
CPTAC	Proteomic	NCI	Proteomic (protein/phosphoprotein) data linked to TCGA tumor samples.

Moreover, developing versatile GRN inference methods to use multiple sources of data whenever available is important for improving GRN inference because multiple complementary data can provide more insights into underlying gene regulatory mechanisms. However, integrating multiple modalities of data (e.g. multi-omics data) to infer GRNs is still a major challenge in the field.

## Gold standard datasets for training and testing gene regulatory network inference methods

Obtaining enough high-quality labeled data is critical for training and/or testing machine learning methods to address any scientific problem, including the GRN inference. Below is a summary of the main datasets available for training and testing GRN inference methods.

- DREAM bulk RNA-seq. The DREAM dataset (https://www.synapse.org/syn3049712) supplies bulk RNA-seq profiles—average expression across mixed cells—for benchmarking GRN tools such as ARACNe and GENIE3, which infer edges from co-expression and MI (https://www.synapse.org/Synapse:syn3049712/wiki/74630) [34, 71].
- Single-cell RNA-seq. Zeisel *et al.*’s scRNA-seq data (GSE60361) resolve gene expression per cell, exposing heterogeneity and rare types; methods like SCENIC and PIDC exploit these profiles to build cell specific GRNs (http://www.ncbi.nlm.nih.gov/geo/query/acc.cgi?acc=GSE60361) [55, 100, 101].
- GTEx. The GTEx portal provides tissue resolved expression from healthy donors; PANDA uses these data (https://www.gtexportal.org/home/) to construct tissue specific regulatory networks, clarifying contextdependent gene control [99, 102].
- DREAM4/5 benchmarks. Synthetic and real expression matrices with known edges enable objective evaluation of algorithms such as GENIE3, TIGRESS, and Inferelator.
- ChIP-seq. Genome-wide TF binding maps supply direct regulatory evidence, serving as priors or validation for predicted GRN links.
- Single-cell multi-omics. Datasets that pair modalities (e.g. RNA + ATAC in Buenrostro 2018) let tools like scMTNI integrate layers and uncover hidden regulation (https://github.com/pinellolab/scATACbenchmarking/tree/master/Real_Data/Buenrostro_2018) [103].
- GRNdb. A repository of precomputed RNA-seq–based networks across multiple species, useful for validating new GRN predictions [104].
- Reactome. A curated pathway database whose interaction data, accessed via resources like Pathway Commons and PSIA, enrich GRN inference with biological context [105, 106].
- DoRothEA. Confidence ranked TF–target compendium for human/mouse; models such as VIPEP draw on it to estimate TF activity and refine networks [107].
- TRUST v2. Manually curated TF target interactions (directionality included) provide high quality priors or benchmarks to heighten the biological relevance of inferred GRNs [108].
- KEGG (Kyoto Encyclopedia of Genes and Genomes).
  KEGG offers comprehensive pathway maps that detail molecular interactions and reactions, serving as a widely used reference for annotating genes and proteins [109].
- WikiPathways. This community-maintained database features a diverse collection of curated biological pathways, making it a valuable resource for integrating pathway-level information into GRN inference [110].
- RegulonDB. Focused on *Escherichia coli*, RegulonDB curates detailed information on transcriptional regulation, including binding sites and operon organization, which is essential for constructing accurate regulatory networks [111].

In addition to the GRN datasets above, there is a tool GeneNetWeaver (GNW) [112] that can generate synthetic gene expression data based on known network topologies. It was used in the DREAM challenges to create datasets with known regulatory networks, which was then used to benchmark GRN inference algorithms such as ARACNe, GENIE3, and CLR.

## Evaluation metrics for gene regulatory network inference

Evaluating predicted GRNs is crucial for assessing the accuracy and reliability of inference methods. It helps identify their strengths and weaknesses, providing insights into performance across different contexts. Validation and benchmarking ensure the correctness and robustness of reconstructed GRNs, guiding researchers in selecting the most reliable methods for their studies. This prevents inaccurate reconstructions that could lead to false conclusions or predictions. Below is a list of common metrics for evaluating inferred GRNs.

### Common evaluation metrics

AUROC stands for the area under the receiver operating characteristic curve. It is a metric used to evaluate the accuracy of classification models, such as those used to infer GRNs. AUROC is calculated by plotting the true positive rate (TPR) against the false positive rate (FPR) at different threshold values for the predicted edges (gene regulatory interactions). The area under the resulting curve is then calculated to obtain the AUROC value, which ranges from 0 to 1, with higher values indicating better performance.

AUPRC stands for area under the precision-recall curve. It is also a common metric used to evaluate the accuracy of inferred GRNs. However, AUPRC is calculated by plotting the precision against the recall at different threshold values for the predicted gene regulatory interactions. The area under the resulting curve is then calculated to obtain the AUPRC value, which ranges from 0 to 1. AUPR concentrates more on performance in searching for true positives than AUROC while reducing false positives, making it more informative in GRN inference tasks in comparison to AUROC.

Precision and recall provide granular insights into model performance at a specific threshold. Precision measures the proportion of predicted regulatory interactions that are correct (TP / (TP + FP)), while recall assesses the proportion of actual regulatory interactions that are successfully identified (TP / (TP + FN)). Here TP, FP, and FN denote the number of true positives, false positives, and false negatives, respectively. In GRN inference, there is typically a trade-off between these two metrics: increasing recall can result in more false positives and thus lower precision, and vice versa. These metrics are essential for understanding whether a model favors sensitivity (recall) over specificity (precision) or achieves a balance between the two.

F1 score is the geometric mean of precision and recall (i.e. 2 × precision × recall / (precision + recall)), which combines precision and recall into a single metric that balances the trade-off between the two. It provides a more comprehensive perspective on model accuracy when both metrics are critical. This is particularly useful in GRN inference, where precision and recall are often equally important.

We summarized and listed the GRN methods, the datasets they were evaluated on, and key performance metrics (AUROC, AUPRC, and F1-score) reported in their own publications in Table 3. While not a unified, strict benchmark, this summary provides a useful point of reference for understanding the performance of different approaches within their respective contexts.

**Table 3:** Performance of various GRN inference algorithms in their respective context.

**Algorithm name**	**Dataset**	**AUROC**	**AUPRC**	**F1**
GENIE3	DREAM4 Multifactorial challenge	0.197	0.798	N/A
SIRENE	Dataset for E.coli [72]	$\sim$ 0.9	N/A	N/A
GRADIS	DREAM4	0.86	0.81	N/A
DeepIMAGER	BMM and dendritic cells	0.95	N/A	N/A
STGRNs	Top 500 highly variable genes datasets(mESC-GM)	0.873	0.742	N/A
SPREd	SERGIO-generated dataset	$\sim$ 0.75	N/A	N/A
RSNET	DREAM network(with scale 50)	0.838	N/A	N/A
dynGENIE3	DREAM4(100-gene networks)	0.32	0.48	N/A
GRNFormer	BEELINE	0.9	0.86	0.96
AnomalGRN	TF + 500(hESC network)	0.99	0.98	N/A
GENECI	DREAM3	0.6196	0.2523	N/A
LASSO	DREAM5	$\sim$ 0.64	$\sim$ 0.28	N/A
ARACNE	Synthetic dataset	N/A	N/A	N/A
MRNET	SynTReN1	0.65	0.1	N/A
CLR	GEO(GSE44770)	0.95	N/A	N/A
GRN-VAE	BEELINE dataset	N/A	N/A	N/A
DeepMAPS	DoRothEA	N/A	N/A	$\sim$ 0.13
GRGNN	DREAM5	0.903	N/A	N/A
DeepMCL	mHSC scRNA-seq	0.93	N/A	N/A
GCLink	TFs + 1000 (mHSC-E)	0.904	0.91	N/A

Table 4 provides a qualitative analysis of the advantages and disadvantages of each GRN inference method, offering practical insight into their use cases, limitations, and computational requirements.

**Table 4:** Advantages and disadvantages of different GRN inference methods.

**Algorithm name**	**Advantage**	**Disadvantage**
GENIE3	Non-parametric, no kinetic assumptions	High computational cost; accuracy collapses if TF identities are unknown
SIRENE	Trains rapidly via per-TF SVMs	Sensitivity falls sharply with few known TF–target pairs
GRADIS	Captures global topology via graph-distance features	Prone to overfitting and instability on small or highly imbalanced datasets
DeepIMAGER	Captures complex nonlinear and combinatorial signals	Very high compute cost
STGRNs	Largelyinsensitive tohyperparameter tweaks	Relies heavily on labeled data
SPREd	No separate feature-importance aggregation steps required	Synthetic training data may not generalize to real-world datasets
RSNET	Infers directed edges and captures both linear and nonlinear dependencies	Recursive optimization adds computational overhead
dynGENIE3	Supports joint steady-state/time-series inference	Real-data performance is inconsistent
GRNFormer	Generalizable across species and celltypes with robust performance	Complexity of the methodology
AnomalGRN	Tackles positive/negative link imbalance by treating true edges as anomalies	Depends heavily on scRNA-seq data quality
GENECI	Outstanding robustness with clear generalization	Heavy computational cost
LASSO	Fuses networks across conditions for more accurate, stable inference	Requires careful tuning of multiple regularization parameters
ARACNE	Prunes indirect correlations to cut false positives and boost biological credibility	May remove true feed-forward or multi-step links, lowering recall in complex networks
MRNET	Reduces redundant MI to cut noise and sharpen feature rankings	Only pairwise MI—misses complex interactions and underperforms on small cohorts
CLR	Infers directed networks via DDPI using only expression data	Depends on imputed expression
GRN-VAE	Uses dropout augmentation to boost robustness against scRNA-seq sparsity	Sensitive to noise level; requires careful tuning of augmentation ratio
DeepMAPS	Interpretable multi-omics network and celltype inference	Scales poorly to very large datasets
GRGNN	Fuses multiple heuristic skeletons, graph embeddings, and expression features in a motif-aware GNN	Only benchmarked on E.coli and yeast; real-world generalization is unproven
DeepMCL	Multi-view contrastive learning integrates diverse co-expression contexts	High computational cost
GCLink	Supports few-shot generalization	Relies on a well-annotated source for pretraining; may underperform with sparse labels

### Evaluation framework and benchmark

Rigorously and objectively evaluating GRN inference methods is important and challenging. Standard evaluation frameworks and benchmarks can streamline the evaluation process and make it easier for users and developers alike to evaluate GRN inference methods. BEELINE is a systematic framework developed to evaluate the accuracy of the methods that infer GRNs from single-cell gene expression data [113]. It uses synthetic networks with predictable cellular trajectories, curated Boolean models, as well as different real-experimental datasets along with ground truth labels for evaluating the accuracy of GRN inference algorithms. BEELINE aids in evaluating GRNs by providing a strategy to simulate single-cell gene expression data from these two types of networks that avoid the pitfalls of previously used methods. The framework also provides recommendations to users of GRN inference algorithms, including suggestions on how to create simulated gene expression datasets for testing them. BEELINE is available at http://github.com/murali-group/BEELINE under an open-source license and will aid in the future development of GRN inference algorithms for single-cell transcriptomic data.

Additionally, GRNbenchmark [114] is a web server for rigorous evaluation of GRN inference methods, offering diverse datasets with varying noise levels, interactive summary plots, and downloadable accuracy metrics. GeneRNIB [115] complements this with a dynamic framework grounded in context-specific evaluation, continuous integration of new algorithms and data, and eight novel causal-inference metrics applied across 10 state-of-the-art methods and multiple omics datasets. Together, these platforms overcome the biases of traditional benchmarks and foster objective, reproducible, and context-aware GRN method development.

## Challenges and future direction

Despite the significant progress made by machine learning methods above, there are several limitations and challenges in the field of GRN inference. The first major challenge is that there is a lack of standard method (like AlphaFold [116] for protein structure prediction) that can generally make high-accuracy GRN inference for different cells and different species in different biological conditions. A tool can only reasonably capture one or a few aspects of a GRN for some cells in some conditions. No method can always outperform others in inferring putative transcriptional targets, putative post-translational targets, or master regulators that drive certain phenotypes [113]. Therefore, it is important to develop sophisticated AI methods that can generalize well to all kinds of real-world biological environment. Mimicking how deep learning has revolutionized protein structure prediction, one direction is to develop more sophisticated deep learning methods such as transformers and diffusion models [117] that are suitable for representing multiple sources of omics data and the interactions between them to accurately infer GRNs in different biological contexts, regardless of species and cells. Simply applying an off-shelf deep learning method to GRN inference will unlikely yield optimal results. The advanced deep learning methods specially customized for GRN inference like AlphaFold2 and AlphaFold3 specially designed for protein sequence and structures are needed to improve the accuracy of GRN inference across the board.

An emerging avenue in addressing these challenges is the integration of foundation models into GRN inference. Foundation models, which are large pretrained neural networks that capture broad representations from massive datasets, have demonstrated exceptional performance in natural language processing and computer vision [118]. By fine-tuning such models on domain-specific data have shown that they can effectively extract meaningful biological insights even from complex omics datasets. In the context of GRN inference, foundation models could be adapted to learn representations that capture the intricate relationships among genes, TFs, and regulatory elements. This approach not only leverages vast amounts of heterogeneous data but also allows for more flexible model-based inference, where the model’s learned representations can be used as priors to improve the inference of regulatory networks. The development of such models promises to mitigate issues related to data sparsity and heterogeneity while providing uncertainty estimates that enhance the reliability of the inferred networks.

The second major challenge is to integrate multi-omics data, particularly, increasingly popular single-cell multi-omics (sc-Multi-omics) data i.e. very sparse and of high-dimensionality, which makes it difficult to identify meaningful patterns and relationships between genes. Another difficulty is the heterogeneity of scMulti-omics data, which may contain different types of cells with distinct gene expression profiles and regulatory mechanisms. Furthermore, GRN inference from scMulti-omics data requires the integration of multiple types of omics data, such as scRNA-seq and scATAC-seq, which may have different levels of noise and bias. Integrating these different types of omics data is difficult due to technical limitations and differences in experimental protocols. Moreover, there is a need for accurate cell clustering to identify cell-type-specific gene expression profiles and regulatory mechanisms. However, accurate cell clustering can be difficult to achieve due to noise, batch effects, and other confounding factors in scMulti-omics data. Therefore, GRN inference from scMulti-omics data requires advanced and robust AI methods that can handle large-scale datasets with high dimensionality and complexity, addressing the issues related to data quality, heterogeneity, integration, cell clustering accuracy, and computational efficiency [119]. This call for the development of more innovative AI methods, particularly deep learning models like contrastive learning based multi-modal AI models like CLIP [120] for text, image, and video processing, to tackle this challenge. Contrastive learning provides an effective, self-supervised approach for multi-omics integration. Table 5 provides a concise comparison of four major learning paradigms for GRN inference—supervised, unsupervised, semi-supervised, and contrastive learning—by listing each paradigm’s principal advantages and disadvantages.

**Table 5:** Comparison of four learning paradigms for GRN inference.

**Learning type**	**Advantages**	**Disadvantages**
Supervised	High predictive accuracy for TF–gene pairs. Leverages prior biological knowledge (e.g. ChIP-seq). Handles heterogeneous features such as expression and epigenetic data.	Requires large labeled interaction sets. Biased toward known edges; may miss novel regulatory relationships. Risk of overfitting when labels or noise are imbalanced.
Unsupervised	Does not require any labeled TF–target pairs. Scalable to genome-wide analyses via correlation or mutual information. Can reveal novel co-regulatory modules without annotation bias.	High false-positive rate since expression ≠ direct regulation. Sensitive to noise and batch effects. Limited interpretability; modules may not map cleanly to TF–target relationships.
Semi-supervised	Combines a small set of labeled interactions with abundant unlabeled data. Reduces labeling cost by propagating information via graph-based frameworks. Supports weak or partial supervision.	Depends heavily on quality of initial labeled seeds; noisy seeds propagate errors. Requires careful tuning of labeled versus unlabeled loss terms. Higher algorithmic complexity when optimizing over both labeled and unlabeled data.
Contrastive	Learns robust gene/node embeddings by contrasting positive and negative pairs. Integrates multi-omics signals (e.g. scRNA-seq, ATAC-seq) for richer representations. Reduces reliance on curated labels by generating positives/negatives via augmentations.	Computationally intensive; requires large batches and many epochs. Sensitive to choice of positive/negative pair generation; risk of collapse. Emerging in GRN inference with fewer established benchmarks.

The third major challenge is the lack of reliable real ground–truth GRNs against which to training and evaluate GRN inference methods. Despite there are some ground-truth networks available (see Section 4), the amount of data is still very limited and not sufficient to train GRN inference methods that can generalize well to different biological conditions, considering the complexity of GRN inference. Moreover, the existing ground-truth networks are usually incomplete and miss many regulatory interactions, making it hard to train and test GRN inference methods. Due to this problem, simulated data have been widely used to assess the performance of network inference methods. However, these simulated data sets may not always accurately represent the real-world GRNs [121] and cannot substitute the real-world GRN data. One way to tackle this challenge is to extract more ground-truth GRNs from biomedical literature. Sophisticated large language models (LLMs) such as ChatGPT may be able to help automate this process to some degree upon well-designed prompts. Therefore, how to design prompts for LLMs to accurately retrieve known GRNs buried in the literature can be an interesting direction to pursue. Moreover, creating a central database to store all the known GRNs and the corresponding input data like the Protein Data Bank (PDB) for protein structure is also important to enable the machine learning and AI community to develop sophisticated GRN inference methods. Future research can explore prompt design and fine-tuning strategies for LLMs to accurately retrieve and integrate known GRNs from the literature, ultimately contributing to the creation of such a central, comprehensive.

Finally, most existing methods focus on inferring static GRNs, even though GRNs dynamic changes in cells in response to internal and external stimuli. It is still very challenging to infer dynamic GRNs [122]. Current methods lack flexibility when it comes to specifying when and under what conditions an interaction between two proteins or a TF and its targets is likely to be realized. To advance solutions to this problem, more dynamic GRN data need to be collected and the AI methods that can track the dynamics of biological systems, like the ones of tracking objects and inferring actions in videos, need to be developed for GRN inference. AI agents that can conduct a series of reasoning and inference according to external inputs may also be applied to infer dynamic GRNs.

**Key Points**

- A comprehensive and in-depth review of machine learning methods, particularly recent deep learning methods, for gene regulatory network (GRN) inference and modeling is presented.
- A new taxonomy of GRN inference methods is provided.
- The resources for training and testing GRN methods are surveyed.
- Major challenges in GRN inference are identified and potential directions to address them are discussed.

## Data Availability

All data referenced are publicly available in the cited literature.

## Notes

**Biographical Note:** The University of Missouri–Columbia, founded in 1839, is a leading public research university known for excellence in education, innovation, and outreach, serving Missouri and the global community.

## References

[1] Wollheim FA . Molecular biology of the cell. Alberts B, Johnson A, Lewis J, Raff M, Roberts K, Walter P (eds), Garland Science, 2002, published price 44£ sterling, weight 3,130 kg, illustrated ISBN 0‐8153‐3218‐1. Scandinavian Journal of Rheumatology. 2003;32:125-.

[2] Lambert SA, Jolma A, Campitelli LF. et al. The human transcription factors. Cell 2018;172:650–65. 10.1016/j.cell.2018.01.029 29425488 PMC12908702

[3] Vonesch SC, Lamparter D, Mackay TFC. et al. Genome-wide analysis reveals novel regulators of growth in Drosophila melanogaster. PLoS Genet 2016;12:e1005616. 10.1371/journal.pgen.1005616 26751788 PMC4709145

[4] Davidson EH, Levin M. Gene regulatory networks. Proc Natl Acad Sci 2005;102:4935–8. 10.1073/pnas.0502024102 15809445 PMC556010

[5] Levine M . Transcriptional enhancers in animal development and evolution. Curr Biol 2010;20:R754–63. 10.1016/j.cub.2010.06.070 20833320 PMC4280268

[6] Spitz F, Furlong EE. Transcription factors: From enhancer binding to developmental control. Nat Rev Genet 2012;13:613–26. 10.1038/nrg3207 22868264

[7] Barabasi AL, Oltvai ZN. Network biology: Understanding the cell's functional organization. Nat Rev Genet 2004;5:101–13. 10.1038/nrg1272 14735121

[8] Aalto A, Viitasaari L, Ilmonen P. et al. Gene regulatory network inference from sparsely sampled noisy data. Nat Commun 2020;11:3493. 10.1038/s41467-020-17217-1 32661225 PMC7359369

[9] Huynh-Thu VA, Sanguinetti G (2019). Gene regulatory network inference: An introductory survey. In: Sanguinetti G, Huynh-Thu V (eds), Gene Regulatory Networks. Methods in Molecular Biology, 2019, vol 1883. Humana Press, New York, NY. 10.1007/978-1-4939-8882-2_1 30547394

[10] Davidson EH, Levine MS. Properties of developmental gene regulatory networks. Proc Natl Acad Sci U S A 2008;105:20063–6. 10.1073/pnas.0806007105 19104053 PMC2629280

[11] Green PJ, Kay SA, Lam E. et al. *In vitro* DNA footprinting. In: Gelvin SB, Schilperoort RA, Verma DPS (eds) Plant Molecular Biology Manual, 1989. Springer, Dordrecht. 10.1007/978-94-009-0951-9_21

[12] Schena M, Shalon D, Davis RW . et al. Quantitative monitoring of gene expression patterns with a complementary DNA microarray. Science. 1995;270:467–70. 10.1126/science.270.5235.467 7569999

[13] Hager GL, Elbi C, Johnson TA. et al. Chromatin dynamics and the evolution of alternate promoter states. Chromosome Research. 2006;14:107–16. 10.1007/s10577-006-1030-0 16506100

[14] Hellman LM, Fried MG. Electrophoretic mobility shift assay (EMSA) for detecting protein-nucleic acid interactions. Nat Protoc 2007;2:1849–61. 10.1038/nprot.2007.249 17703195 PMC2757439

[15] Mortazavi A, Williams BA, McCue K. et al. Mapping and quantifying mammalian transcriptomes by RNA-Seq. Nat Methods 2008;5:621–8. 10.1038/nmeth.1226 18516045 PMC13303166

[16] Karlebach G, Shamir R. Modelling and analysis of gene regulatory networks. Nat Rev Mol Cell Biol 2008;9:770–80. 10.1038/nrm2503 18797474

[17] Chen G, Ning B, Shi T. Single-cell RNA-Seq technologies and related computational data analysis. Front Genet 2019;10:317. 10.3389/fgene.2019.00317 31024627 PMC6460256

[18] Trapnell C . Defining cell types and states with single-cell genomics. Genome Res 2015;25:1491–8. 10.1101/gr.190595.115 26430159 PMC4579334

[19] Liu ET, Pott S, Huss M. Q&a: ChIP-seq technologies and the study of gene regulation. BMC Biol 2010;8:56. 10.1186/1741-7007-8-56 20529237 PMC2871264

[20] Buenrostro JD, Wu B, Chang HY. et al. ATAC-seq: A method for assaying chromatin accessibility genome-wide. Curr Protoc Mol Biol 2015;109:21.29.1–9. 10.1002/0471142727.mb2129s109 PMC4374986 25559105

[21] Wu J, Zhao X, Lin Z. et al. Large scale gene regulatory network inference with a multi-level strategy. Mol Biosyst 2016;12:588–97. 10.1039/C5MB00560D 26687446

[22] Park S, Kim JM, Shin W. et al. BTNET: Boosted tree based gene regulatory network inference algorithm using time-course measurement data. BMC Syst Biol 2018;12:69–77. 10.1186/s12918-018-0547-0 29560827 PMC5861501

[23] Huynh-Thu VA . Machine Learning-Based Feature Ranking: Statistical Interpretation and Gene Network Inference PhD thesis,. Liège, Belgium: Université de Liège, 2012.

[24] Angelini C, Costa V. Understanding gene regulatory mechanisms by integrating ChIP-seq and RNA-seq data: Statistical solutions to biological problems. Frontiers in cell and developmental biology 2014;2:51. 10.3389/fcell.2014.00051 25364758 PMC4207007

[25] Clark SJ, Lee HJ, Smallwood SA. et al. Single-cell epigenomics: Powerful new methods for understanding gene regulation and cell identity. Genome Biol 2016;17:72. 10.1186/s13059-016-0944-x 27091476 PMC4834828

[26] Michael B, Eisen Paul T, Spellman Patrick O. et al. Cluster analysis and display of genome-wide expression patterns. Proc Natl Acad Sci U S A 1998;95:14863–8. 10.1073/PNAS.95.25.14863 9843981 PMC24541

[27] Chai LE, Loh SK, Low ST. et al. A review on the computational approaches for gene regulatory network construction. Comput Biol Med 2014;48:55–65. 10.1016/j.compbiomed.2014.02.011 24637147

[28] Mochida K, Koda S, Inoue K. et al. Statistical and machine learning approaches to predict gene regulatory networks from transcriptome datasets. Front Plant Sci 2018;9:1770. 10.3389/fpls.2018.01770 30555503 PMC6281826

[29] Zhu X, Huang Q, Luo J. et al. Mini-review: Gene regulatory network benefits from three-dimensional chromatin conformation and structural biology. Computational and Structural Biotechnology Journal. 2023;21:1728–37. 10.1016/j.csbj.2023.02.028 PMC9986247 36890880

[30] Jiang T, Gradus JL, Rosellini AJ. Supervised machine learning: A brief primer. Behav Ther 2020;51:675–87. 10.1016/j.beth.2020.05.002 32800297 PMC7431677

[31] Razaghi‐Moghadam Z, Nikoloski Z. Supervised learning of gene regulatory networks. Current protocols in plant biology. 2020;5:e20106. 10.1002/CPPB.20106 32207875

[32] Maetschke SR, Madhamshettiwar PB, Davis MJ. et al. Supervised, semi-supervised and unsupervised inference of gene regulatory networks. Brief Bioinform 2014;15:195–211. 10.1093/bib/bbt034 23698722 PMC3956069

[33] Yang B, Bao W, Chen B. et al. Single_cell_GRN: Gene regulatory network identification based on supervised learning method and Single-cell RNA-seq data. BioData Mining 2022;15:13. 35690842 10.1186/s13040-022-00297-8 PMC9188720

[34] Huynh-Thu VA, Irrthum A, Wehenkel L. et al. Inferring regulatory networks from expression data using tree-based methods. PloS One 2010;5:e12776. 10.1371/journal.pone.0012776 20927193 PMC2946910

[35] Huynh-Thu VA, Geurts P. dynGENIE3: Dynamical GENIE3 for the inference of gene networks from time series expression data. Sci Rep 2018;8:3384. 10.1038/s41598-018-21715-0 29467401 PMC5821733

[36] Petralia F, Wang P, Yang J. et al. Integrative random forest for gene regulatory network inference. Bioinformatics 2015;31:i197–205. 10.1093/bioinformatics/btv268 26072483 PMC4542785

[37] Saremi M, Amirmazlaghani M. Reconstruction of gene regulatory networks using multiple datasets. IEEE/ACM Trans Comput Biol Bioinform 2021;19:1827–39. 10.1109/TCBB.2021.3057241 33539303

[38] Zhang Y, Chen Q, Gao D. et al. “GRRFNet: Guided Regularized Random Forest-based Gene Regulatory Network Inference Using Data Integration,” 2020 IEEE International Conference on Bioinformatics and Biomedicine (BIBM), Seoul, Korea (South), 2020, pp. 132–139, 10.1109/BIBM49941.2020.9313349

[39] Haury AC, Mordelet F, Vera-Licona P. et al. TIGRESS: Trustful inference of gene regulation using stability selection. BMC Syst Biol 2012;6:1–17. 10.1186/1752-0509-6-145 23173819 PMC3598250

[40] Awad, Mamoun, Latifur Khan. “Support Vector Machines.” In Intelligent Information Technologies: Concepts, Methodologies, Tools, and Applications, Vijayan Sugumaran (ed.), 1138–1146. Hershey, PA: IGI Global Scientific Publishing, 2008. 10.4018/978-1-59904-941-0.ch065

[41] Pavlidis P, Weston J, Cai J et al. Gene functional classification from heterogeneous data. In: Proceedings of the fifth annual international conference on Computational biology (RECOMB '01). New York, NY, USA: Association for Computing Machinery, 2001, 249–255. 10.1145/369133.369228

[42] Mordelet F, Vert JP. SIRENE: Supervised inference of regulatory networks. Bioinformatics 2008;24:i76–82. 10.1093/bioinformatics/btn273 18689844

[43] Gillani Z, Akash MSH, Rahaman MM. et al. CompareSVM: Supervised, support vector machine (SVM) inference of gene regularity networks. BMC bioinformatics 2014;15:1–7. 10.1186/s12859-014-0395-x 25433465 PMC4260380

[44] Razaghi-Moghadam Z, Nikoloski Z. Supervised learning of gene-regulatory networks based on graph distance profiles of transcriptomics data. NPJ systems biology and applications 2020;6:21. 10.1038/s41540-020-0140-1 32606380 PMC7327016

[45] Ni Y, Aghamirzaie D, Elmarakeby H. et al. A machine learning approach to predict gene regulatory networks in seed development in Arabidopsis. Front Plant Sci 2016;7:1936. 10.3389/fpls.2016.01936 28066488 PMC5179539

[46] Aluru M, Shrivastava H, Chockalingam SP. et al. EnGRaiN: A supervised ensemble learning method for recovery of large-scale gene regulatory networks. Bioinformatics 2022;38:1312–9. 10.1093/bioinformatics/btab829 34888624

[47] Jiang X, Zhang X. RSNET: Inferring gene regulatory networks by a redundancy silencing and network enhancement technique. BMC bioinformatics 2022;23:165. 10.1186/s12859-022-04696-w 35524190 PMC9074326

[48] Tjärnberg A, Morgan DC, Studham M. et al. GeneSPIDER–gene regulatory network inference benchmarking with controlled network and data properties. Mol Biosyst 2017;13:1304–12. 10.1039/C7MB00058H 28485748

[49] Tjärnberg A, Nordling TE, Studham M. et al. Avoiding pitfalls in L 1-regularised inference of gene networks. Mol Biosyst 2015;11:287–96. 10.1039/C4MB00419A 25377664

[50] Tjärnberg A, Nordling TE, Studham M. et al. Optimal sparsity criteria for network inference. J Comput Biol 2013;20:398–408. 10.1089/cmb.2012.0268 23641867

[51] Hillerton T, Seçilmiş D, Nelander S. et al. Fast and accurate gene regulatory network inference by normalized least squares regression. Bioinformatics 2022;38:2263–8. 10.1093/bioinformatics/btac103 35176145 PMC9004640

[52] Friedman J, Hastie J, Tibshirani R . Regularization Paths for Generalized Linear Models via Coordinate Descent. J Stat Softw 2010;33:1–22. 10.18637/jss.v033.i01 PMC2929880 20808728

[53] LeCun Y, Bengio Y, Hinton G. Deep learning. Nature 2015;521:436–44. 10.1038/nature14539 26017442

[54] Zhou X, Pan J, Chen L. et al. DeepIMAGER: Deeply Analyzing Gene Regulatory Networks from scRNA-seq Data. Biomolecules. 2024;14:7. 10.3390/biom14070766 PMC11274664 39062480

[55] Chan TE, Stumpf MPH, Babtie AC. Gene regulatory network inference from single-cell data using multivariate information measures. Cell Syst 2017;5:251–267.e253. 10.1016/j.cels.2017.08.014 28957658 PMC5624513

[56] Matsumoto H, Kiryu H, Furusawa C. et al. SCODE: An efficient regulatory network inference algorithm from single-cell RNA-Seq during differentiation. Bioinformatics 2017;33:2314–21. 10.1093/bioinformatics/btx194 28379368 PMC5860123

[57] Kim S . Ppcor: An R package for a fast calculation to semi-partial correlation coefficients. Commun Stat Appl Methods 2015;22:665–74. 10.5351/CSAM.2015.22.6.665 26688802 PMC4681537

[58] Papili Gao N, Ud-Dean SM, Gandrillon O. et al. SINCERITIES: inferring gene regulatory networks from time-stamped single cell transcriptional expression profiles. Bioinformatics. 2018;34:258–66. https://pubmed.ncbi.nlm.nih.gov/28968704/10.1093/bioinformatics/btx575 PMC5860204 28968704

[59] Wu Z, Sinha S. SPREd: a simulation-supervised neural network tool for gene regulatory network reconstruction, Bioinformatics Advances, 2024;4:vbae011. 10.1093/bioadv/vbae011 PMC10913396 38444538

[60] Slawek J, Arodz T. ENNET: Inferring large gene regulatory networks from expression data using gradient boosting. BMC Syst Biol 2013;7:106. 10.1186/1752-0509-7-106 24148309 PMC4015806

[61] Passemiers A, Moreau Y, Raimondi D. Fast and accurate inference of gene regulatory networks through robust precision matrix estimation. Bioinformatics 2022;38:2802–9. 10.1093/bioinformatics/btac178 35561176 PMC9113237

[62] Vaswani A, Shazeer N, Parmar N. et al. Attention is all you need. Advances in neural information processing systems. 2017;30.

[63] Shaw P, Uszkoreit J, Vaswani A. Self-attention with relative position representations. arXiv preprint arXiv:1803.02155. 2018 Mar 6.

[64] Fuhr J. Benefits and limits of advanced methods used for transformer diagnostics. In 2009 IEEE Electrical Insulation Conference 2009 May 31 (pp. 262–272). IEEE, 10.1109/EIC.2009.5166355

[65] Xu J, Zhang A, Liu F. et al. STGRNS: an interpretable transformer-based method for inferring gene regulatory networks from single-cell transcriptomic data. Bioinformatics. 2023;39:btad165. 10.1093/bioinformatics/btad165 PMC10085635 37004161

[66] Hegde A, Cheng J. GRNFomer: Accurate Gene Regulatory Network Inference Using Graph Transformer. bioRxiv. 2025:2025–01. 10.1101/2025.01.26.634966 PMC13069479 41883144

[67] Zhou Z, Wei J, Liu M. et al. AnomalGRN: deciphering single-cell gene regulation network with graph anomaly detection. BMC biology. 2025;23:73. 10.1186/s12915-025-02177-z PMC11900578 40069807

[68] Fogel DB. Evolutionary computation: toward a new philosophy of machine intelligence. John Wiley & Sons; 2006. 10.1002/0471749214

[69] Segura-Ortiz A, García-Nieto J, Aldana-Montes JF. et al. GENECI: a novel evolutionary machine learning consensus-based approach for the inference of gene regulatory networks. Computers in Biology and Medicine. 2023;155:106653. 10.1016/j.compbiomed.2023.106653 36803795

[70] Zheng L, Tian C. Information Theory and Machine Learning. MDPI-Multidisciplinary Digital Publishing Institute; 2022.

[71] Margolin AA, Nemenman I, Basso K. et al. ARACNE: An algorithm for the reconstruction of gene regulatory networks in a mammalian cellular context. BMC Bioinformatics 2006;7:S7. 10.1186/1471-2105-7-S1-S7 PMC1810318 16723010

[72] Faith JJ, Hayete B, Thaden JT. et al. Large-scale mapping and validation of *Escherichia coli* transcriptional regulation from a compendium of expression profiles. PLoS Biol 2007;5:e8. 10.1371/journal.pbio.0050008 17214507 PMC1764438

[73] Gan Y, Hu X, Zou G. et al. Inferring gene regulatory networks from single-cell transcriptomic data using bidirectional RNN. Frontiers in Oncology. 2022;12:899825. 10.3389/fonc.2022.899825 PMC9178250 35692809

[74] Kingma DP, Welling M. An introduction to variational autoencoders. Foundations and Trends® in Machine Learning. 2019;12:307–92.

[75] Zhou J, Troyanskaya OG. Predicting effects of noncoding variants with deep learning–based sequence model. Nature methods. 2015;12:931–4. 10.1038/nmeth.3547 PMC4768299 26301843

[76] Ma A, Wang X, Li J. et al. Single-cell biological network inference using a heterogeneous graph transformer. Nat Commun 2023;14:964. 10.1038/s41467-023-36559-0 36810839 PMC9944243

[77] Friedman N, Linial M, Nachman I. et al. Using Bayesian networks to analyze expression data. J Comput Biol 2000;7:601–20. 10.1089/106652700750050961 11108481

[78] Zhou J, Cui G, Zhang Z. et al. Graph neural networks: A review of methods and applications. AI Open 2020;1:57–81. 10.1016/j.aiopen.2021.01.001

[79] Liu W, Teng Z, Li Z. et al. CVGAE: A Self-Supervised Generative Method for Gene Regulatory Network Inference Using Single-Cell RNA Sequencing Data. Interdisciplinary Sciences: Computational Life Sciences. 2024;16:990–1004. 10.1007/s12539-024-00633-y 38778003

[80] Bansal M, Belcastro V, Ambesi-Impiombato A. et al. How to infer gene networks from expression profiles. Mol Syst Biol 2006;2:2006. 10.1038/msb4100074 PMC1828749 17299415

[81] Larranaga P, Kuijpers CM, Murga RH. et al. Genetic algorithms for the traveling salesman problem: A review of representations and operators. Artificial Intelligence Review 1999;13:129–70. 10.1023/A:1006529012972

[82] Daoudi M, Meshoul S, Boucherkha S. A semi-supervised approach to GRN inference using learning and optimization. InResearch Anthology on Bioinformatics, Genomics, and Computational Biology 2024 (pp. 94-118). IGI Global Scientific Publishing.

[83] Wang J, Ma A, Ma Q. et al. Inductive inference of gene regulatory network using supervised and semi-supervised graph neural networks. Comput Struct Biotechnol J. 2020;18:3335–43. 33294129 10.1016/j.csbj.2020.10.022 PMC7677691

[84] den Oord V. Representation learning with contrastive predictive coding. arXiv e-prints. 2018:arXiv.

[85] Chen T, Kornblith S, Norouzi M. et al. A simple framework for contrastive learning of visual representations. In: *International Conference on Machine Learning* 2020 Nov 21 (pp. 1597–1607). PmLR.

[86] Khosla P, Teterwak P, Wang C. et al. Supervised contrastive learning. Advances in Neural Information Processing Systems. 2020;33:18661–73.

[87] Lin Z, Ou-Yang L. Inferring gene regulatory networks from single-cell gene expression data via deep multi-view contrastive learning. Brief Bioinform 2023;24:bbac586. 10.1093/bib/bbac586 36585783

[88] Yu M, Zhang H, Xu L. GCLink: A graph contrastive link prediction framework for gene regulatory network inference. BMC Bioinformatics 2025;26:45. 39960893 10.1093/bioinformatics/btaf074 PMC11881698

[89] Bonneau R, Reiss DJ, Shannon P. et al. The Inferelator: An algorithm for learning parsimonious regulatory networks from systems-biology data sets de novo. Genome Biol 2006;7:R36. 10.1186/gb-2006-7-5-r36 16686963 PMC1779511

[90] Meyer PE, Kontos K, Lafitte F. et al. Information-theoretic inference of large transcriptional regulatory networks. EURASIP Journal on Bioinformatics and Systems Biology 2007;2007:79879. 10.1155/2007/79879 18354736 PMC3171353

[91] Shannon P, Markiel A, Ozier O. et al. Cytoscape: A software environment for integrated models of biomolecular interaction networks. Genome Res 2003;13:2498–504. 10.1101/gr.1239303 14597658 PMC403769

[92] ENCODE Project Consortium . An integrated encyclopedia of DNA elements in the human genome. Nature 2012;489:57–74. 10.1038/nature11247 22955616 PMC3439153

[93] Roadmap Epigenomics Consortium, et al. Integrative analysis of 111 reference human epigenomes. Nature 2015;518:317–30. 10.1038/nature14248 25693563 PMC4530010

[94] Wilhelm M, Schlegl J, Hahne H. et al. Mass-spectrometry-based draft of the human proteome. Nature 2014;509:582–7. 10.1038/nature13319 24870543

[95] Stuart T, Butler A, Hoffman P. et al. Comprehensive integration of single-cell data. Cell 2019;177:1888–1902.e21. 10.1016/j.cell.2019.05.031 31178118 PMC6687398

[96] Argelaguet R, Arnol D, Bredikhin D. et al. MOFA+: A statistical framework for comprehensive integration of multi-modal single-cell data. Genome Biol 2020;21:111. 10.1186/s13059-020-02015-1 32393329 PMC7212577

[97] Butler A, Hoffman P, Smibert P. et al. Integrating single-cell transcriptomic data across different conditions, technologies, and species. Nat Biotechnol 2018;36:411–20. 10.1038/nbt.4096 29608179 PMC6700744

[98] Argelaguet R, Velten B, Arnol D. et al. Multi-omics factor analysis—A framework for unsupervised integration of multi-omics data. Mol Syst Biol 2018;14:e8124. 10.15252/msb.20178124 29925568 PMC6010767

[99] Glass K, Huttenhower C, Quackenbush J. et al. Passing messages between biological networks to refine predicted interactions. PloS One 2013;8:e64832. 10.1371/journal.pone.0064832 23741402 PMC3669401

[100] Aibar S, González-Blas CB, Moerman T. et al. SCENIC: Single-cell regulatory network inference and clustering. Nat Methods 2017;14:1083–6. 10.1038/nmeth.4463 28991892 PMC5937676

[101] Zeisel A, Muñoz-Manchado AB, Codeluppi S. et al. Cell types in the mouse cortex and hippocampus revealed by single-cell RNA-seq. Science. 2015;347:1138–42. 10.1126/science.aaa1934 25700174

[102] The GTEx Consortium . The genotype-tissue expression (GTEx) pilot analysis: Multitissue gene regulation in humans. Science 2015;348:648–60. 10.1126/science.1262110 25954001 PMC4547484

[103] Zhang S, Pyne S, Pietrzak S. et al. Inference of cell type-specific gene regulatory networks on cell lineages from single cell omic datasets. Nature Communications. 2023;14:3064. 10.1038/s41467-023-38637-9 PMC10224950 37244909

[104] Paytuvi-Gallart A. et al. A gene regulatory network atlas for *Arabidopsis thaliana*. Front Genet 2020;11:468.32477409

[105] Fabregat A, Jupe S, Matthews L. et al. The Reactome pathway knowledgebase. Nucleic Acids Res 2018;46:D649–55. 10.1093/nar/gkx1132 29145629 PMC5753187

[106] Tarca AL, Draghici S, Khatri P. et al. A novel signaling pathway impact analysis. Bioinformatics 2009;25:75–82. 10.1093/bioinformatics/btn577 18990722 PMC2732297

[107] Garcia-Alonso L, Holland CH, Ibrahim MM. et al. Benchmark and integration of resources for the estimation of human transcription factor activities. Genome Res 2019;29:1363–75. 10.1101/gr.240663.118 31340985 PMC6673718

[108] Han H, Cho JW, Lee S. et al. TRUST v2: An expanded reference database of human and mouse transcriptional regulatory interactions. Nucleic Acids Res 2018;46:D380–6. 10.1093/nar/gkx1013 29087512 PMC5753191

[109] Kanehisa M, Sato Y, Furumichi M. et al. New approach for understanding genome variations in KEGG. Nucleic Acids Res 2019;47:D590–5. 10.1093/nar/gky962 30321428 PMC6324070

[110] Slenter DN, Kutmon M, Hanspers K. et al. WikiPathways: A multifaceted pathway database bridging metabolomics to other omics research. Nucleic Acids Res 2018;46:D661–7. 10.1093/nar/gkx1064 29136241 PMC5753270

[111] Santos-Zavaleta A, Salgado H, Gama-Castro S. et al. RegulonDB v 10.5: Tackling challenges to unify classic and high throughput knowledge of gene regulation in *Escherichia coli*. Nucleic Acids Res 2019;47:D212–20. 10.1093/nar/gky1077 30395280 PMC6324031

[112] Schaffter T, Marbach D, Floreano D. GeneNetWeaver: in silico benchmark generation and performance profiling of network inference methods. Bioinformatics 2011;27:2263–70. 10.1093/bioinformatics/btr373 21697125

[113] Pratapa A, Jalihal AP, Law JN. et al. Benchmarking algorithms for gene regulatory network inference from single-cell transcriptomic data. Nat Methods 2020;17:147–54. 10.1038/s41592-019-0690-6 31907445 PMC7098173

[114] Seçilmiş D, Hillerton T, Sonnhammer ELL. GRNbenchmark*—*a web server for benchmarking directed gene regulatory network inference methods. Nucleic Acids Res 2022;50:W398–404. 10.1093/nar/gkac377 35609981 PMC9252735

[115] geneRNIB . A living benchmark for gene regulatory network inference Jalil Nourisa, Antoine Passemiers, Marco stock, Berit Zeller-Plumhoff, Robrecht Cannoodt, Christian Arnold, Alexander Tong, Jason Hartford, Antonio Scialdone, Yves Moreau, Yang Li, Malte D. Luecken bioRxiv 2025;02:640181. 10.1101/2025.02.25.640181

[116] Jumper J, Evans R, Pritzel A. et al. Highly accurate protein structure prediction with AlphaFold. Nature 2021;596:583–9. 10.1038/s41586-021-03819-2 34265844 PMC8371605

[117] Guo Z, Liu J, Wang Y. et al. Diffusion models in bioinformatics and computational biology. Nature Reviews Bioengineering 2024;2:136–54. 10.1038/s44222-023-00114-9 PMC10994218 38576453

[118] Bommasani R. On the opportunities and risks of foundation models. arXiv preprint arXiv:2108.07258. 2021.

[119] Kim D, Tran A, Kim HJ. et al. Gene regulatory network reconstruction: Harnessing the power of single-cell multi-omic data. NPJ Systems Biology and Applications 2023;9:51. 37857632 10.1038/s41540-023-00312-6 PMC10587078

[120] Ventre E, Herbach U, Espinasse T. et al. One model fits all: Combining inference and simulation of gene regulatory networks. PLoS Comput Biol 2023;19:e1010962. 10.1371/journal.pcbi.1010962 36972296 PMC10079230

[121] Radford A, Kim JW, Hallacy C. et al. Learning transferable visual models from natural language supervision. In: International Conference on Machine Learning 2021 Jul 1 (pp. 8748-8763). PmLR.

[122] Mousavi R, Konuru SH, Lobo D. Inference of dynamic spatial GRN models with multi-GPU evolutionary computation. Brief Bioinform 2021;22:bbab104. 33834216 10.1093/bib/bbab104

